# Terpinen-4-ol: A Novel and Promising Therapeutic Agent for Human Gastrointestinal Cancers

**DOI:** 10.1371/journal.pone.0156540

**Published:** 2016-06-08

**Authors:** Shiran Shapira, Shlomo Pleban, Diana Kazanov, Peter Tirosh, Nadir Arber

**Affiliations:** 1 Laboratory of Molecular Biology, The Integrated Cancer Prevention Center, Tel Aviv Sourasky Medical Center, affiliated to the Sackler Faculty of Medicine, Tel Aviv University, Tel-Aviv, Israel; 2 EMERALD BIO-LABS LTD, Netanya, Israel; Baylor University Medical Center, UNITED STATES

## Abstract

**Background:**

Terpinen-4-ol, a naturally occurring monoterpene is the main bioactive component of tea-tree oil and has been shown to have many biological activities.

**Aim:**

To study the antitumor effects of terpinen-4-ol and its mechanism of action in prostate and GI malignancies, alone and in combination with chemotherapeutic and biological agents.

**Methods:**

Terpinen-4-ol was administrated alone or combined with standard chemotherapy (Oxaliplatin, Fluorouracil, Gemcitabine, Tarceva) and biological agent (Cetuximab). It was also combined with humanized anti-CD24 mAbs (was developed by us). Killing effects were measured qualitatively by light microscopy and quantitatively using the MTT and FACS analysis, following treatment of colorectal, pancreatic, gastric and prostate cancer cells. Terpinen-4-ol effect on tumor development was evaluated in xenograft model.

**Results:**

Terpinen-4-ol induces a significant growth inhibition of colorectal, pancreatic, prostate and gastric cancer cells in a dose-dependent manner (10–90% in 0.005–0.1%). Terpinen-4-ol and various anti-cancer agents (0.2μM oxaliplatin and 0.5μM fluorouracil) demonstrated a synergistic inhibitory effect (83% and 91%, respectively) on cancer cell proliferation. In *KRAS* mutated colorectal cancer cells, which are resistant to anti-EGFR therapy, combining of terpinen-4-ol with cetuximab (1 μM) resulted in impressive efficacy of 80–90% growth inhibition. Sub-toxic concentrations of terpinen-4-ol potentiate anti-CD24 mAb (150μg/ml)-induced growth inhibition (90%). Considerable reduction in tumor volume was seen following terpinen-4-ol (0.2%) treatment alone and with cetuximab (10mg/kg) (40% and 63%, respectively) as compare to the control group.

**Conclusion:**

Terpinen-4-ol significantly enhances the effect of several chemotherapeutic and biological agents. The possible molecular mechanism for its activity involves induction of cell-death rendering this compound as a potential anti-cancer drug alone and in combination in the treatment of numerous malignancies. Terpinen-4-ol restores the activity of cetuximab in cancers with mutated KRAS.

## Introduction

Essential oils and their components extracted from vegetable materials have been found to exhibit anti-microbial, anti-viral, anti-fungal, anti-oxidant, anti-inflammatory and anti-cancer activities [1–3].

Monoterpenes are major plant-derived secondary metabolites widely found in natural products, including fruits, vegetables and herbs and known to be associated with the plant defense mechanisms. The monoterpenes consist of two isoprene units, and are found in large amounts in essential oils [4,5]. In addition, many monoterpenes have been proposed to exert potent anticancer activity. Some of them reportedly displayed promising results in the prevention and treatment of different types of leukemia and cancers, such as breast, skin, pancreatic and colon cancers in rodents [6]. Notably, several of these compounds, among them Perillyl alcohol and limonene, are being testing in ongoing human studies [7–9].

Terpinen-4-ol, one of the primary active ingredients of the tea tree oil, consists of a mixture of more than 100 different compounds, and is found in a variety of aromatic plants (oranges, mandarins, origanum, New Zealand lemonwood tree, Japanese cedarand black pepper) [10]. Terpinen-4-ol is a potent bactericidal agent [11] that possess antifungal properties [12]. Of particular interest is its *in vitro* activity against *Staphylococcus aureus* and *C*. *albicans*[13,14]. It was shown that combining this natural substance and conventional drugs may help treat resistant yeast and bacterial infections.

Several recent reports have suggested that terpinen-4-ol induces antitumor effects by selectively causing necrotic cell death and cell-cycle arrest in melanoma cell lines, or by triggering caspase-dependent apoptosis in human melanoma cells, particularly in drug (Adriamycin) resistant cells [15,16]. Moreover, terpinen-4-ol was shown to elicit a dose-dependent cytotoxic response on human non-small cell lung cancer cells, presumably through the involvement of the mitochondrial apoptotic pathway [17].

CD24 is a small, heavily glycosylated mucin-like cell surface protein anchored to the membrane via glycosyl phosphatidylinositol (GPI)[18]. CD24 is known to be overexpressed in various human malignancies, both solid and hematological [19], and is usually tied with a more aggressive course of the disease [18,20,21]. We have shown that anti-CD24-based cancer immunotherapy has potential clinical application in solid tumors [21–23]. Therefore, the combination of terpinen-4-ol together with anti-CD24 therapy was evaluated in this work.

In this study, we aimed to show the anticancer effects of terpinen-4-ol in various types of cancer cells *in vitro* and *in vivo*. It is also shown that Terpinen-4-ol can restore the potency of cetuximab in tumors with a mutant RAS.

## Materials and Methods

### Materials

All reagents were purchased from Sigma, Israel unless otherwise stated. Cell culture media and additives were obtained from Beit-Haemek, Israel. Annexin V and propidium iodide were purchased from Biotium.

The following materials were tested:

1. Terpinene-4-ol
2. Mixture 1 = γ-terpinene, α-terpinene, 1,8-cineole, p-cymene, terpinene-4-ol
3. Mixture 2 = γ-terpinene, α-terpinene, 1,8-cineole, p-cymene,

The ratio of each compound is described in Table 1.

**Table 1:** 

Name	Mix 1	Mix 2
Terpinene-4-ol	4.0gr	—
γ-terpinene	2.0gr	4.0gr
α-terpinene	1.0gr	1.0gr
1,8-cineole	0.5gr	2.0gr
p-cymene	0.5gr	2.0gr
Ethanol Absolute	2.0gr	2.0gr

The following chemotherapy drugs and antibodies were tested:

1. ElOXATIN^®^ (Oxaliplatin) at a concentrations ranging between 0.2–0.5 μM.
2. 5-FU (Fluorouracil)- 0.3–0.5 μM.
3. GEMZAR (Gemcitabine)- 0.1–1 μM
4. Tarceva (0.05–0.1 μM).
5. Erbitux (Cetuximab) at a concentration range of 1 μM.
6. Humanized anti-CD24 mAb (humanized IgG1 antibody that binds to the cell surface CD24 protein) at concentrations of 75–150 μg/ml.

### Cell lines

Human CRC (HT29, HCT116, COLO320), gastric carcinoma (AGS), pancreas (COLO357, Panc-1, MIA-PACA) cells lines were grown in high-glucose Dulbecco’s modified Eagle’s medium (DMEM) supplemented with 5% heat-inactivated (HI) fetal bovine serum (FBS), 1% glutamine and streptomycin/penicillin. An androgen-independent prostate (DU145, CL-1), and colorectal DLD1 cancer cells were grown in RPMI-1640 medium supplemented with 5% HI-FBS.

### MTT cell viability assay

Cells were seeded in 96-well plates (1x10^4^ cells/well) in complete medium. On the following day, different concentrations of the above-described agents were added to the cells in triplicates. At 72 h later, the medium was replaced by fresh media (100 μl per well) containing 1 mg/ml 3-(4,5-dimethylthiazol-2-yl)-2,5-diphenyltetrazolium bromide (MTT) and incubated for 2–4 h. MTT-formazan crystals were dissolved by the addition of 100 μl extraction buffer. Absorbance at 570nm and a reference wavelength of 690nm were recorded on an automated microplate reader. The relative number of viable cells was compared to untreated cells.

### Transfection and luciferase assay

Transfections were performed using jetPEI^™^ (Polyplus-transfection Inc, NY, USA) according to the manufacturer's instructions. 5 x 10^5^HCT116 cells were seeded in 6-well plates for Luc assays. The next day, when the cells were about 50% confluent, co-transfection with 3 μg of PY4-SV40-LUC vector plus 0.3 ng of pRL-CMV (Promega) was performed. 24h after the transfection medium was replaced and the cells were exposed to cetuximab, different concentration of terpinen-4-ol or left untreated. Luc assay was performed 48 h post after the treatment. Briefly, cells were washed once with PBS and then lyzed in 250 ~μl of lysis buffer for 5 min at room temperature. The lysates were centrifuged at 14,000 rpm for 5 min, and 20 μl of each lysate were used to measure Luc reporter gene expression. The Luc activity was normalized to Renilla Luc activity from a parallel co-transfection of pRL-CMVDual Luc system, Promega).

### Detection of cell death

Cells were seeded in 12-well plates (1x10^5^ cells/well) in complete medium and treated with terpinen-4-ol at several concentrations for 72 h. Annexin V was detected according to the manufacturer’s protocol. The cells were washed with PBS and then incubated in a solution of the membrane-impermeable nuclear dye propidium iodide. The cells were then immediately analyzed by flow cytometry [FACSCalibur (Becton Dickinson, CA)], and the results were analyzed with the CELLQuest program (Becton Dickinson).

### Xenograft model for measuring *in vivo* anti-tumor activity of terpinen-4-ol alone and in combination with biological agents

Male athymic nude mice, 6–8 weeks old, (Harlan Laboratories) were housed in sterile cages and handled with aseptic precautions. They were fed ad libitum. For testing the therapeutic potential of terpinen-4-ol, exponentially growing cancer cells were harvested and resuspended at a final concentration of 5x10^6^ cells per 0.1 ml PBS per injection. The cells were injected subcutaneously into the flank of the mice. When tumors were palpable (~0.3 cm^3^), the mice were randomly divided into groups and the treatment was started (intraperitoneal and/or intratumoral injections). The animals were treated twice a week for 3 weeks. They were weighed and tumor volume was measured with a caliper and plotted every 3 days starting from the onset the treatment. Tumor volume was calculated as 4/3π∙a∙b^2^. At the end of the experiment, the mice were anesthetized and sacrificed by cervical dislocation and the tumors were excised.

### Statistics

Data from the *in vitro* studies are presented as mean±SD of sets of data as determined in triplicates. Statistical significance between treatments was determined by Student’s t-test, and *P* values < .05 were considered significant. In the *in* vivo studies, the tumor-bearing mice were randomized into 5 treatment groups and the tumor volumes were periodically monitored and calculated as 4/3π∙a∙b^2^. Significant differences between groups and at different time points were determined by Student’s t-test.

### Study approval

The study was approved by the institutional committee for animal welfare at Tel-Aviv Sourasky Medical Center.

## Results

### Identification of terpinen-4-ol as the effective ingredient

**Fig 1:**
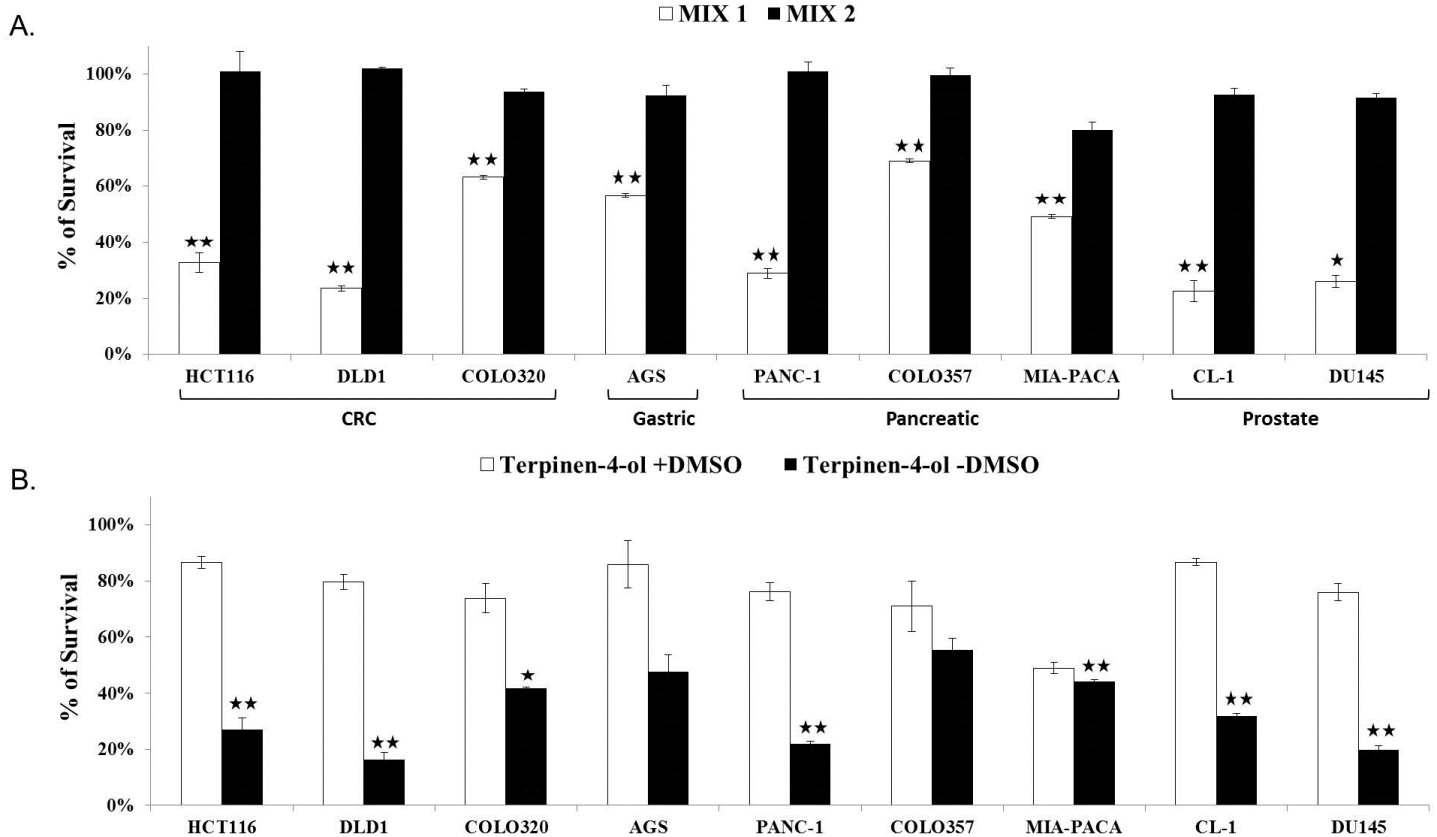
**A. Identification and isolation of terpinen-4-ol.** Two mixtures (their composition is described in Table 1) of monoterpens were tested on colorectal (HCT116, DLD1, COLO320), gastric (AGS), pancreatic (PANC-1, COLO357, MIA-PACA) and prostate (CL-1, DU145) cancer cell lines. 1x10^4^ cells were seeded in 96-well plates in complete medium. 0.05% of the mixtures were added to the cells on the next day. Cell survival was evaluated by enzymatic MTT assay72 h after the treatment. Each bar represents the mean±SD of a set of data determined in triplicates. *P < .*05*, **P < .*005*. **B. Terpinen-4-ol activity does not depend on the presence of DMSO.** 1x10^4^CRC, gastric, pancreatic and prostate cancer cells were seeded in 96-well plates in complete medium.0.05% of terpinen-4-ol with or without 0.01% DMSO was added to the cells on the next day. Cell survival was evaluated by enzymatic MTT assay72 h after the treatment. Each bar represents the mean±SD of a set of data determined in triplicates. *P < .*05*, **P < .*005*.

Two mixtures with different monoterpens composition were tested (Table 1). Mixture 1 was significantly more effective and toxic than mixture 2 (*P<0*.*005*). The main difference between the two was terpinen-4-ol. The results (Fig 1A) indicated for significant differences in cell survival between the mixtures, allowing us to identify terpinen-4-ol and its contribution to the cytotoxic effect.

### No effect of DMSO on the activity of terpinen-4-ol

One of the obstacles that is encountered when using cytotoxic compounds as therapeutic agents is their low solubility in pharmaceutically solutions and their low ability to penetrate into cells. To that extent, we examined whether terpinen-4-ol will be active in the absence of dimethyl sulfoxide (DMSO). DMSO was used to dissolve terpinen-4-ol and its effect was evaluated. It emerged that the cytotoxic activity of terpinen-4-ol was not hampered by adding DMSO (Fig 1B). Several concentrations of terpinen-4-ol (0.005%-0.1%) with and without DMSO were tested (data not shown) and the difference in the effect of DMSO was observed mainly at higher doses of terpinen-4-ol. DMSO seemed interfere or inhibit the biological effect of terpinen-4-ol, maybe by hindering the absorption of this biologically active molecule.

### Terpinen-4-ol as an anti-cancer agent

**Fig 2:**
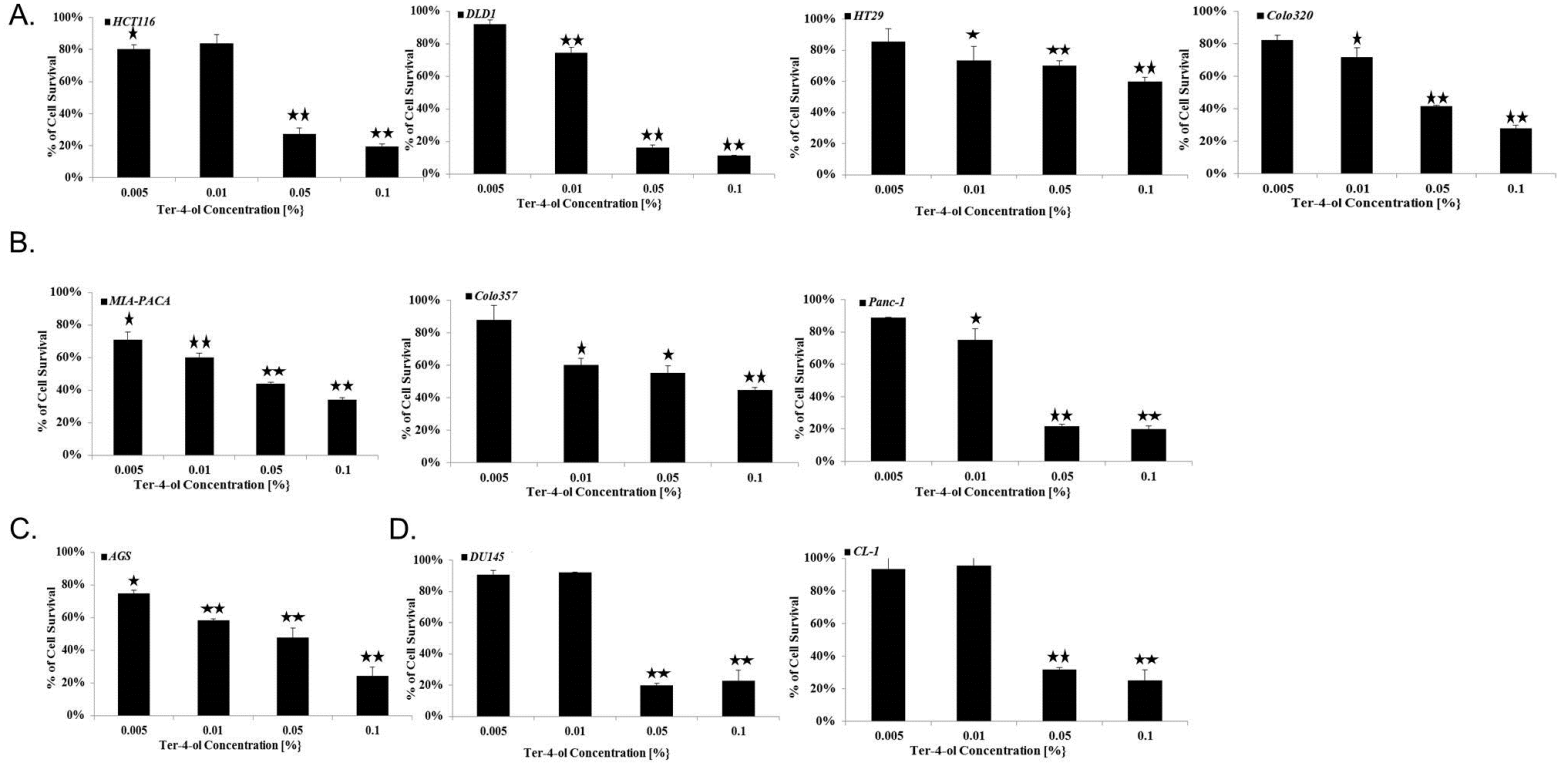
Terpinen-4-ol can act as an anticancer agent. 1x10^4^ HCT116, DLD1, HT29 and Colo320 CRC (A), MIA-PACA, Colo357 and Panc-1 pancreatic (B), AGS gastric (C), and DU145 and CL-1 prostate cancer cells (D) were seeded in 96-well plates in complete medium. Several concentrations of terpinen-4-ol [0.005%, 0.01%, 0.05 and 0.1% (v/v)] were added to the cells on the next day. Cell survival was evaluated by enzymatic MTT assay 72 h after the treatment. Each bar represents the mean±SD of a set of data determined in triplicates. *P < .*05*, **P < .*005*

Terpinen-4-ol inhibited the growth of colorectal (Fig 2A), pancreatic (Fig 2B), gastric (Fig 2C) and prostate (Fig 2D) cancers in a dose-dependent fashion [10% (in 0.005%)-90 (in 0.1%) growth inhibition)], as measured qualitatively by microscopic observations (data not shown) and quantitatively by the enzymatic MTT assay (Fig 2).

### Terpinen-4-ol induces apoptosis and not necrosis

**Fig 3:**
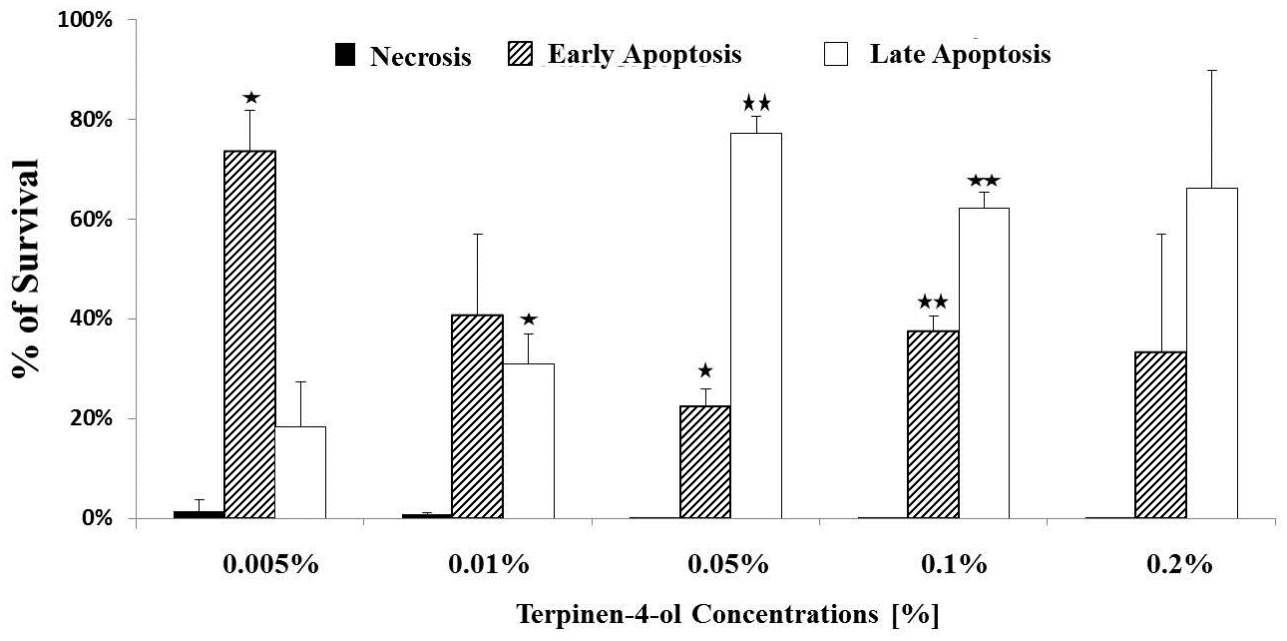
Terpinen-4-ol induces apoptosis in human CRC cells. 1x10^5^ HCT116 cells were seeded in 12-well plates in complete medium and exposed to different concentrations of terpinen-4-ol for 72 h. Cell death was measured by FACS after staining with Annexin V and PI dyes. Each bar represents the mean±SD of a set of data determined in triplicates. *P < .*05*, **P < .*005*

Low concentrations of terpinen-4-ol (0.005–0.01%) inhibited HCT116 cell proliferation in a modest way (20–30%) as measured by the MTT viability assay. High concentrations (0.05–0.1%), induced massive cell death (75–90%). As can be seen in Fig 3, apoptosis is the cell death mechanism responsible for the cytotoxic effect induced by Terpinen-4-ol. Early apoptotic death was induced by low dose of terpinen-4-ol, whereas the percentages of late apoptosis increased at higher concentrations. No necrotic cells were observed.

### Enhancement of terpinen-4-ol efficacy

**Fig 4:**
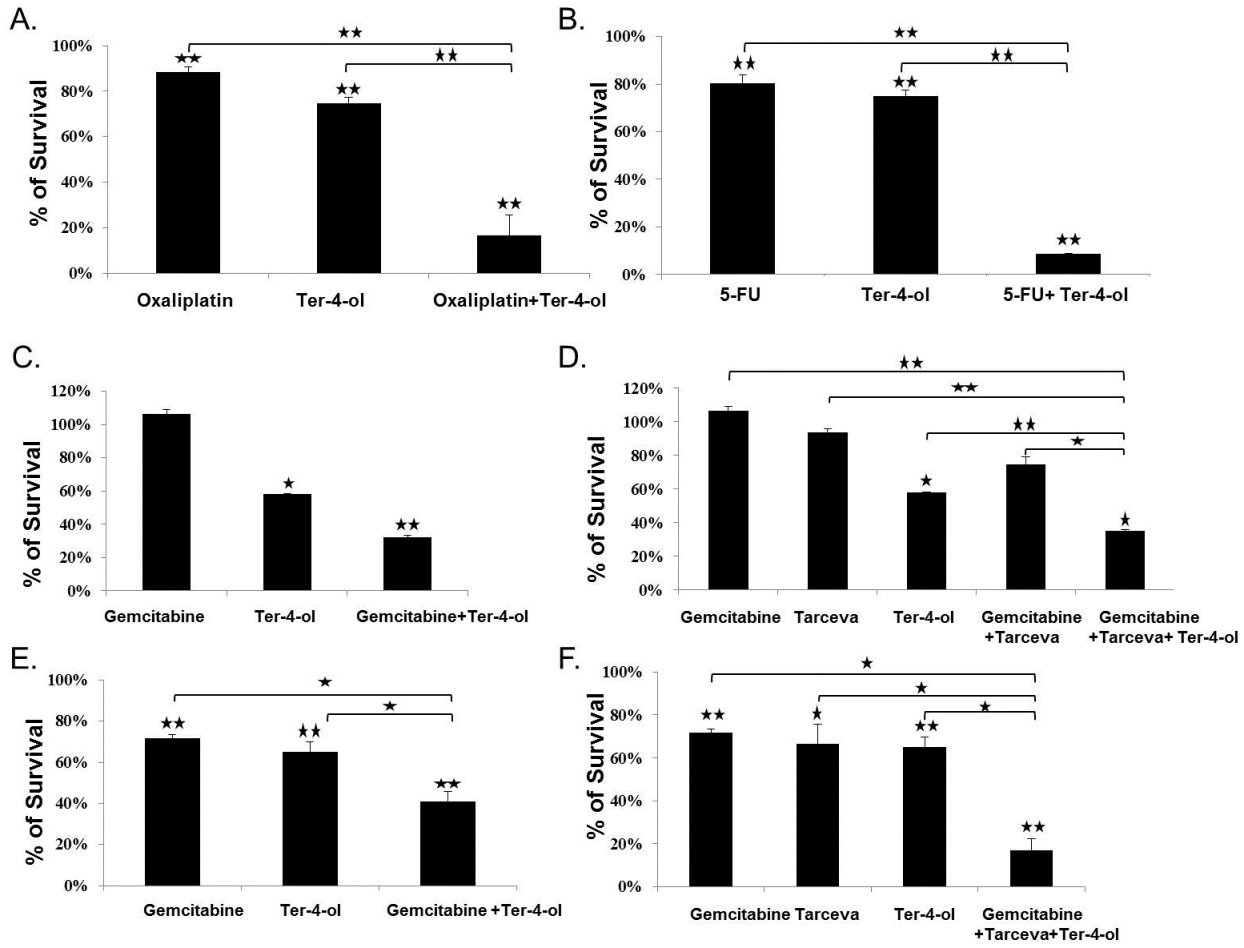
Enhanced cytotoxicity of the combinations ofterpinen-4-ol and different conventional chemotherapy approaches. 1x10^4^ CR DLD1 cells (A,B), and pancreatic MIA-PACA (C,D) and Panc-1 (E,F) cells were seeded in 96-well plates in complete medium. On the next day, oxaliplatin at a concentration of 0.2μM and terpinen-4-ol at a concentration of 0.01% (A), 5-FU at a concentration of 0.3μM and terpinen-4-ol at a concentration of 0.01% (B), gemcitabine at a concentration of 0.1μM and terpinen-4-ol at a concentration of 0.01%, (C) gemcitabine at a concentration of 0.1μM, erlotinib hydrochlorides (Tarceva^®^) at a concentration of 0.1μM, and terpinen-4-ol at a concentration of 0.01% (D), gemcitabine at a concentration of 1μM and terpinen-4-ol at a concentration of 0.01% (E), gemcitabine at a concentration of 1μM, anderlotinib hydrochlorides (Tarceva^®^) at a concentration of 0.1μM, and terpinen-4-ol at a concentration of 0.01% (F) were added for 72 h. Cell survival was evaluated by the enzymatic MTT assay. Each bar represents the mean±SD of a set of data determined in triplicates. *P < .*05*, **P < .*05*

**Fig 5:**
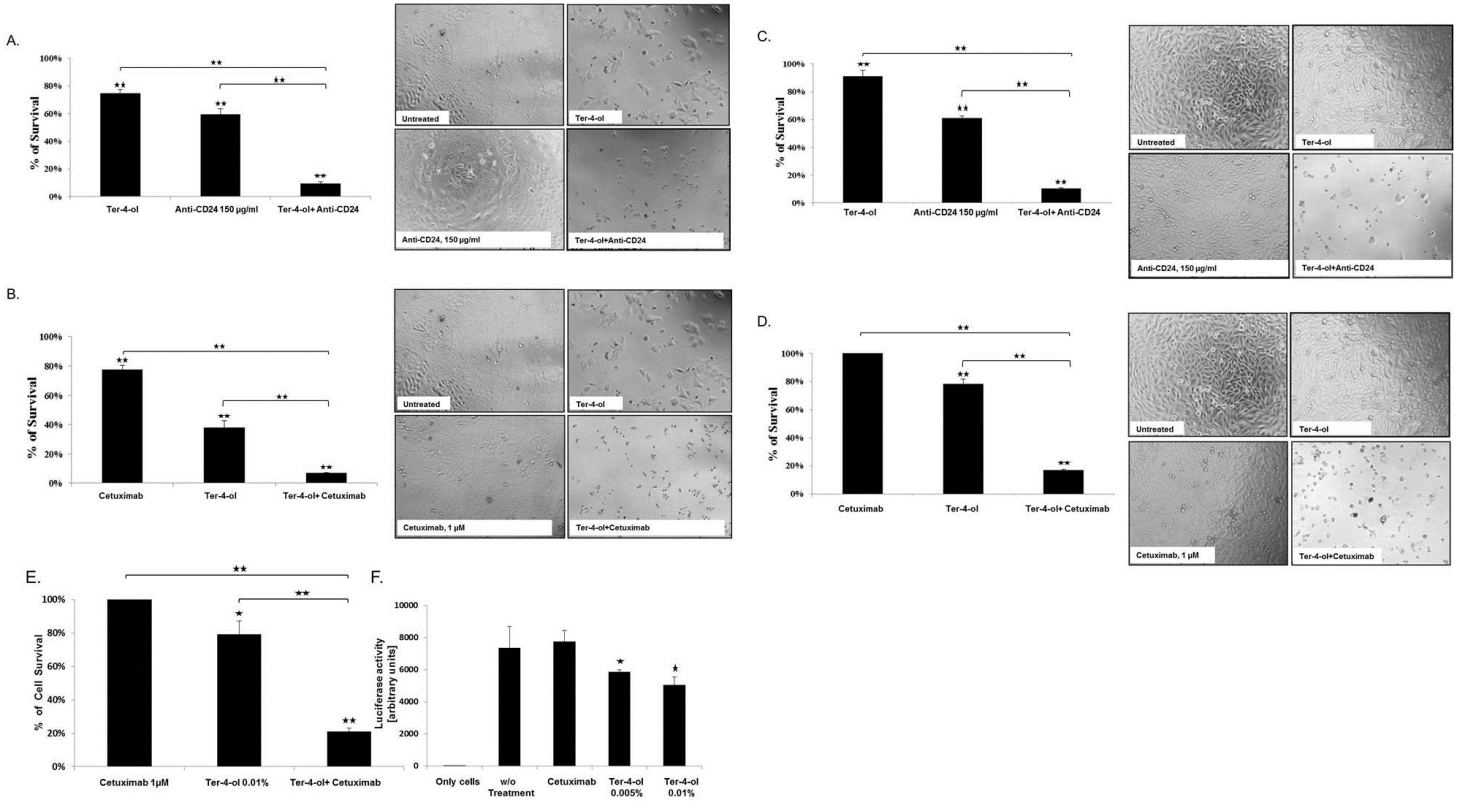
Enhanced cytotoxicity of the combination between terpinen-4-ol and biological treatment tools. 1x10^4^ CR DLD1 and HCT116 cells (A, B, E), and prostate CL-1 (C, D) cells were seeded in 96-well plates in complete medium. On the next day, terpinen-4-ol at a concentration of 0.01% and the humanized anti-CD24 antibodies (150μg/ml) (A, C), and terpinen-4-ol at a concentration of 0.01% and cetuximab at a concentration of 1μM (B,D,E) were added for 72 h. Cell survival was evaluated by the enzymatic MTT assay. Each bar represents the mean±SD of a set of data determined in triplicates. (F) 0.4X10^6^ HCT116 cells were seeded in 6-well plates in triplicates. The next day, co-transfection with PY4-SV40-LUC vector and pRL-CMV was performed. 24h after the transfection medium was replaced and the cells were exposed to cetuximab, different concentration of terpinen-4-ol or left untreated. Luc assay was performed 48 h post after the treatment. Each bar represents the mean±SD of a set of data determined in triplicates. *P < .*05*, **P < .*05*

Terpinen-4-ol was combined with different types of conventional chemotherapy, depending on the type of cancer being treated. For CRC therapy, terpinen-4-ol was combined with oxaliplatin (Fig 4A) and 5-FU (Fig 4B). An impressive synergistic growth inhibition effect was achieved (83% and 91%, for oxaliplatin and 5-FU, respectively) in the combined regimen as compared to each agent alone [12% (oxaliplatin), 25% (terpinen-4-ol), and 20% (5-FU)]. These results have achieved statistical significance, *P<0*.*005*. Similar results were obtained in pancreatic cancer cells (Fig 4C–4F). Terpinen-4-ol impressively increased the efficacy of gemcitabine and that of Tarceva (60–85% cell death).

### Enhanced cytotoxicity of terpinen-4-ol with biological agents

Combining terpinen-4-ol with either humanized anti-CD24 matured antibody (Arber's lab, Tel Aviv, Israel) or the chimeric anti EGFR antibody (Cetuximab, Merck Serono) resulted in a remarkable synergistic growth inhibition effect (85–90%, *P<0*.*005*) on human CRC (Fig 5A and 5B) and prostate cancer cells (Fig 5C and 5D).

### Terpinen-4-ol restores the sensitivity of *K-ras* mutant cancer cells to ceteximub

The DLD1 CRC cells carry a mutation in the *KRAS* oncogene. Therefore, they are resistant to anti-epidermal growth factor (EGFR) therapy. Combining terpinen-4-ol (0.01%) with cetuximab (1 μM) resulted in a rather impressive efficacy of a 85–90% growth inhibition. These results were confirmed in another *KRAS* mutated CRC cell line (HCT116) (Fig 5E), with an 80% growth inhibition (*P<0*.*005*) for the combined therapy.

### Decreased activity of *K-ras* signaling pathway with terpinen-4-ol

For that purpose we used the (Ets/Ap1)_4_ RAS-responsive element (Py4) construct, which we had previously constructed [24–26]. The activity of the *KRAS* pathway was evaluated in mutated CRC cells (HCT116) (Fig 5F). Transfection with Py4-SV40-Luc activity in the presence of terpinen-4-ol (0.005 and 0.01%) was 1.3 and 1.5 fold lower as compared to the activity in its absence (*P<0*.*05*)_. No effect was observed after exposure to cetuximab (1 μM).

### Inhibition of subcutaneous DLD1 tumor growth in mice by terpinen-4-ol

**Fig 6:**
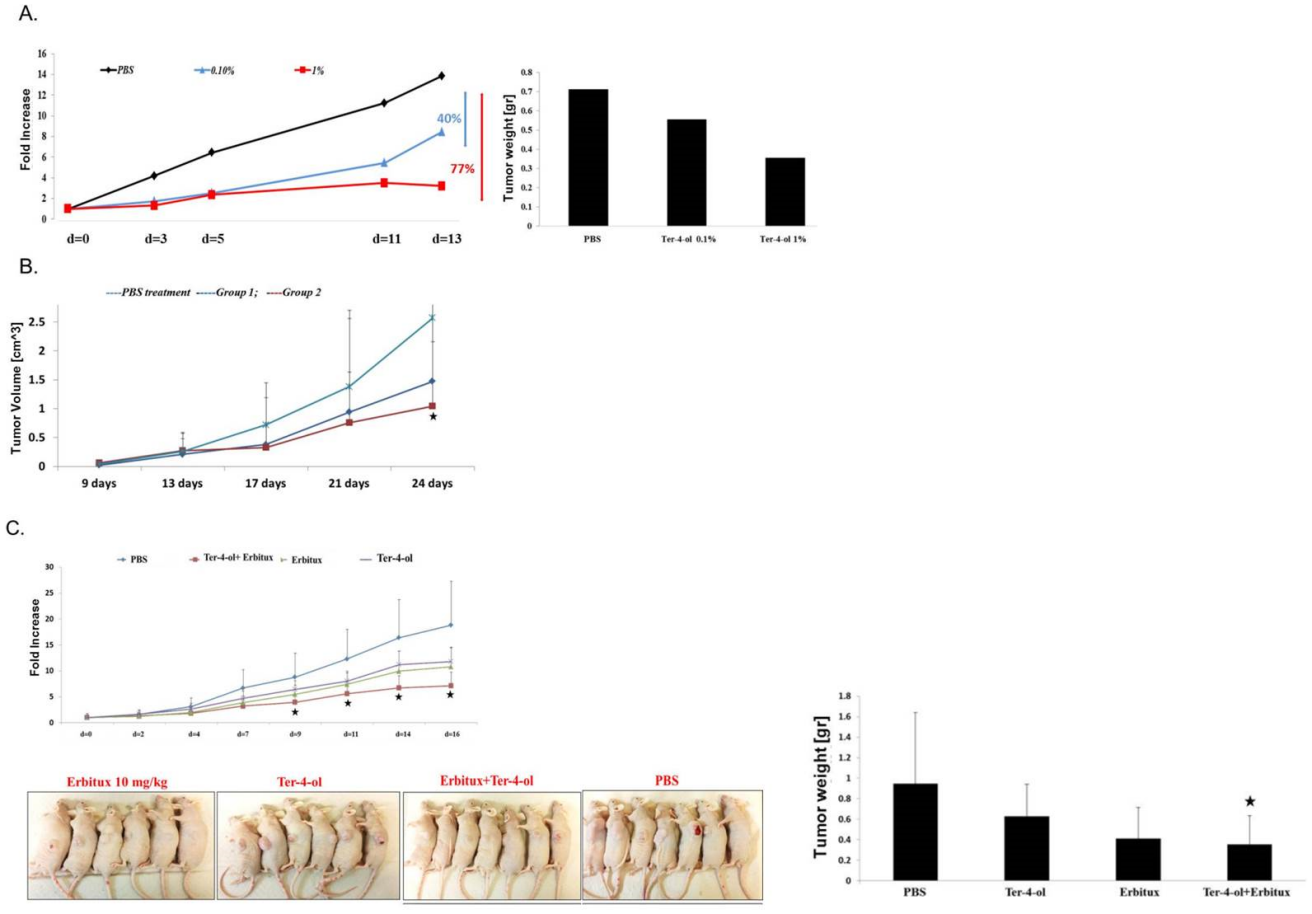
Inhibition of subcutaneous DLD1 tumor growth in mice by terpinen-4-ol. Exponentially growing DLD1 cancer cells were harvested and resuspended at a final concentration of 5x10^6^ cells per 0.1 ml PBS per injection. When the tumors were palpable, the mice were randomly divided into groups and the treatment was started [intratumoral injections of terpinen-4-ol (A, B) or intratumoral injections of terpinen-4-ol and intraperitoneal injections of cetuximab (C)]. The mice were treated twice weekly. They were weighed and tumor volume was measured with a caliper every 3 days starting from the onset of treatment with terpinen-4-ol. The tumor volume vs. time of treatment was plotted. At the end of the experiment, the mice were anesthetized and sacrificed by cervical dislocation and the tumors were excised and measured for volume and weight. Each bar represents the mean±SD. *P < .*05*, **P < .*005*

Next, we tested the potential anti-tumor activity of terpinen-4-ol *in vivo*. Intratumoral injections (5 injections) of the compound (0.1% and 1%) were given twice weekly to nude mice (n = 6) bearing xenografts of CRC DLD1 cells. The treatment was started when tumors were 0.3–0.5 cm^3^. Significant inhibition of tumor development was observed; 40% and 70% reduction in tumor volume and about 25% and 50% reduction of tumor weight (Fig 6A). These results were confirmed in another experiment (Fig 6B). No significant adverse effects were observed (data not shown).

### Combined growth inhibition of subcutaneous tumors in mice

When one of tumor's diameter reached the size of 0.5 mm intratumoral injections of terpinen-4-ol (0.1%) along with systemic (IP) administration of cetuximab (10 mg/kg) resulted in a significant decrease in tumor volume (62% ±2.5%, *P<0*.*05*) and weight (62.5% ±3%, *P<0*.*05*) (Fig 6C)

### Design of a more effective formulation of terpinen-4-ol

**Fig 7:**
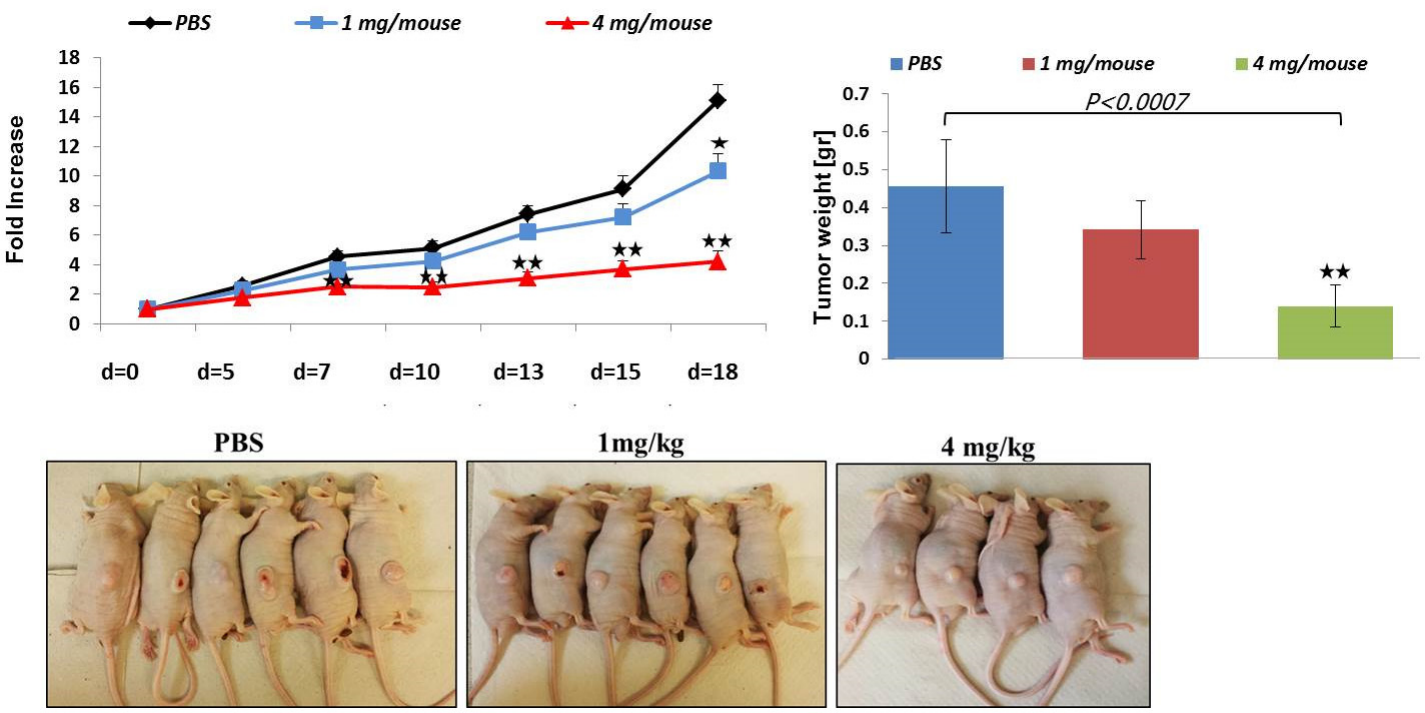
Inhibition of subcutaneous HCT116 tumor growth in mice by formulation of terpinen-4-ol. Exponentially growing HCT116 cancer cells were harvested and resuspended at a final concentration of 5x10^6^ cells per 0.1 ml PBS per injection. When the tumors were palpable, mice were randomly divided into 3groups and the treatment, consisting of intraperitoneal injections of terpinen-4-ol (1 and 4 mg/kg), was started. The mice were treated twice weekly. They were weighed and tumor volume was measured with a caliper every 3 days starting from the onset of treatment with terpinen-4-ol. The tumor volume vs. time of treatment was plotted. At the end of the experiment, the mice were anesthetized and sacrificed by cervical dislocation and the tumors were excised and measured for volume and weight. Each bar represents the mean±SD. *P < .*05*, **P < .*005*

Two different formulations of terpinen-4-ol were evaluated *in vivo*, a nano and suspension formulations. The latter emerged as being more effective and safer (data not shown). Systemic administration of the suspension (1 and 4 mg) to mice bearing xenografts of CRC (HCT116) cells resulted in an impressive decrease in tumor volume (32%±1.1%, *P<0*.*05* and 72%± 0.7%, *P<0*.*005*, respectively) and weight (26% ±1.19 and 70% ± 0.71%, *P<0*.*005*, respectively) in a dose-dependent manner (Fig 7).

## Discussion

The anticancer effects of terpinen-4-ol are impressive in various types of cancer cells both *in vitro* and *in vivo*.

Terpinen-4-ol is a major component of essential oil derived from several aromatic plants. It is used as an anti-inflammatory and antioxidant agent [27–29]. The contribution of terpinen-4-ol as an anti-cancer agent and the underlying signaling pathways of different types of cell death are unknown. Herein, it is shown that the mechanism of action of terpinen-4-ol is induction of apoptosis and not necrosis. It is also shown that terpinen-4-ol and various anticancer agents demonstrate a synergistic growth inhibitory effect by decreasing the survival of various cancer cell lines. Such combinations maybe expected to be more effective and less toxic since lower drug concentrations can be used for treating a wide range of cancers.

Of note, restoring sensitivity to anti-EGFR therapies (e.g. cetuximab), in CRC cases carrying the *KRAS* mutation, when given along with terpinen-4-ol has a merit clinical importance. It is demonstrated by measuring the activity of the ras responsive promoter before and after exposure to terpinen-4-ol. It is shown that the promoter activity was significantly reduced upon exposure to Terpenin-4-ol. This interesting and important observation needs to be confirmed in further laboratory studies before leading to any clinical use of terpinuin-4-ol.

Injection of terpinen-4-ol into the tumor remarkably inhibited tumor growth without any significant adverse effects. In search for more convenient routes of administration, two pharmaceutical formulations were prepared and tested for systemic administration, nano formulation and suspension. Nano formulations increased the surface area and therefore dramatically improved water solubility, bioavailability, effectiveness and efficiency. The suspension form was composed of small drops/molecules of the therapeutically active ingredient (the oil) in a suspension medium. Since the nanodrops were associated with serious toxicity (loss of body weight, mortality), the suspension approach that was devoid of any side effects was chosen for further exploration. The systemic administration of terpinen-4-ol by suspension was associated with a significant reduction in tumor size in the experimental nude mice.

In summary, the use of a combination of plant-derived anticancer substances and chemotherapeutic or biological agents for treating various types of cancer is promising, with a synergetic efficacy that allow a lower concentration of chemotherapy and biological agents that can not only increase efficacy but can minimize toxicity as well. Most importantly terpinen-4-ol major advantage is the capability to restore the sensitivity to EGFR antagonists in tumors with *Ras* mutations.

## Abbreviations

CRC: Colorectal cancer
DMSO: Dimethyl sulfoxide
EGFR: Epidermal growth factor receptor
FACS: Fluorescence-activated cell sorting
Luc: luciferase
